# Progress in pediatrics in 2011. Choices in endocrinology, gastroenterology, hemato-oncology, infectious diseases, otolaryngology, pharmacotherapy and respiratory tract illnesses

**DOI:** 10.1186/1824-7288-38-23

**Published:** 2012-06-08

**Authors:** Carlo Caffarelli, Francesca Santamaria, Silvia Cesari, Angela Di Giorgio, Sergio Bernasconi

**Affiliations:** 1 Department of Pediatrics, University Hospital of Parma, Parma, Italy; 2 Department of Pediatrics, Federico II University, Naples, Italy

**Keywords:** Endocrinology, Gastroenterology, Hemato-oncology, Infectious diseases, Otolaryngology, Pharmacotherapy, Respiratory tract illnesses

## Abstract

Main progresses in endocrinology, gastroenterology, hemato-oncology, infectious diseases, otolaryngology, pharmacotherapy, and respiratory tract illnesses selected from articles published in The Italian Journal of Pediatrics in 2011 were reviewed. Risk factors for gastroenteritis and appendicitis in developing countries may be useful in improving our understanding of these diseases. Childhood hearing impairment is a world-wide problem which continues to have an high prevalence in newborns. Among the mechanisms of diseases, obese children often have asthma and high hepcidin levels that may reduce serum iron concentrations. In cystic fibrosis, 18q distal deletion has been described as a novel mutation. Hypothyroidism in children with central nervous system infections may increase mortality rates. Infrared tympanic thermometer (IRTT) in oral mode for the measurement of body temperature may be useful in fever screening in a busy setup. In newborns, the transmission of CMV infection through breast milk may be prevented through freezing or pasteurization. Recent advances in treatment of constipation, urinary tract infections, leukemia, pain in children with cancer, neonates with sepsis or difficult weaning from mechanical ventilation will likely contribute towards optimizing management of these common disorders. The work of the Family Pediatricians Medicines for Children Research Network aims to develop competence, infrastructure, networking and education for pediatric clinical trials.

## Endocrinology

Obesity has been shown to be associated with low-serum iron concentrations but the etiology of hypoferrinemia still remains unclear. Hepcidin is a small peptide secreted by the liver and adipocytes that has been proposed as a key regulator of iron metabolism. Hepcidin is suppressed in iron deficiency, allowing increased absorption of dietary iron and replenishment of iron stores, while its expression is increased in chronic inflammatory states including obesity. The aim of the study conducted by Sanad et al [1] was to assess the effect of obesity on hepcidin serum levels and its relation to treatment outcome in children with iron deficiency anemia (IDA). Compared to the control group, serum hepcidin was significantly lower in non obese children with IDA and significantly higher in obese children with IDA. Hepcidin increased significantly in non-obese children with IDA after 3 months of oral iron therapy while the obese children did not show a significant change in hepcidin levels after iron therapy. Obesity was therefore associated with higher hepcidin levels and to a diminished response to oral iron therapy in children with iron deficiency anemia.

Only few studies have analysed the changes in thyroid hormones in children affected by central nervous system infections. Jiao and coworkers [2] measured thyroid hormone concentrations in the serum and cerebrospinal fluid of children with CNS infections and compared them with those of children with non-CNS infections, healthy adults, and adults with primary hypothyroidism. Serum T3 and T4 levels were decreased in about half of children with CNS infections, T3 and T4 were much lower than in healthy adults but still higher than in primary hypothyroidism. TSH levels were not significantly different among children with CNS infections and non-CNS infections. Low T3 and T4 levels in serum and cerebrospinal fluid were predominant in children with serious CNS infections. The detection of T3, T4 and TSH through small amounts of blood or cerebrospinal fluid is simple, convenient, and valuable in evaluating thyroid function and prognosis in children with CNS infections, since mortality increases dramatically when thyroxine (T4) falls below certain levels.

## Gastroenterology

Constipation has an high prevalence rate among children. Afzal et al [3] reviewed constipation diagnosis and conducted a systematic review on treatment strategies. They established that disimpaction should be obtained by polyethylene glycol (PEG). More evidence is needed to assess the role of enemas in impaction. Once disimpaction has taken place the aim of laxative treatment should be focused on keeping the child symptom-free with regular soft bowel movements. PEG is the first line treatment. More evidence is needed to assess the role of mineral oil. There was lack of benefit from lactulose, and from senna but more studies are needed.

Diarrhea is one of the major causes of morbidity and mortality among children in developing countries. Aremu et al [4] analyzed the role of socioeconomic status in the treatment of childhood diarrhea in eleven sub-Saharan African countries (Benin, Burkina Faso, Cameroon, Ghana, Kenya, Liberia, Mali, Nigeria, Niger, Senegal, and Tanzania). They showed that poorly educated care-givers and living in socio-economically disadvantaged areas were associated with a lower tendency of using medical centers for the management of diarrhea. These are factors that should be taken into consideration when planning interventions that aim to increase the search for medical care during diarrhea episodes.

Spontaneous bacterial peritonitis is a rather common complication in children with chronic liver disease. El-Shabrawi MHF et al [5] showed that fever is the most helpful for the diagnosis compared to other symptoms or signs. Routine culture of the ascitic fluid was not always positive. Among biochemical parameters, a LDH ascitic/serum ratio ≥ 0.5, an arterial-ascitic pH gradient ≥ 0.1 and total ascitic fluid protein ≤ 1 gm/dl significantly indicated an infection.

Appendicitis is the most common non-traumatic surgical abdominal disorder in children aged 2 years or older. Appendicitis is ultimately diagnosed in 1% to 8% of children who present to paediatric emergency departments with acute abdominal pain. Decreased dietary fiber intake and ingestion of refined carbohydrates represent significant risk factors for appendicitis. Societies with high fiber intake have less than one tenth the incidence of appendicitis compared with areas where fiber intake is lower. The original article from Gardikis and coworkers [6] assesses the incidence and risk factors implicated in acute appendicitis in preschool children in Thrace, Greece. Acute appendicitis in preschoolers developed more frequently in Muslims (39.4%) than in Christians (17.7%).The lack of toilet facilities inside the home, overcrowded living conditions, living in rural areas, and amount of appendix lymphoid tissue were significantly more frequent among the Muslim preschool children. No statistically significant differences between Muslim and Christian children were evidenced regarding gender, family history of acute appendicitis, or vegetable consumption. Authors conclude that a possible explanation for the remarkably higher incidence of acute appendicitis among preschool-aged Muslim children may be the larger amount of lymphoid tissue in the appendix wall combined with a low standard of hygiene.

## Hemato-oncology

Tumor immunotherapy with T lymphocytes, which can recognize and destroy malignant cells, has been limited to the ability of isolating and expanding T cells restricted to tumor-associated antigens. Chimeric antigen receptors (CARs) composed of antibody binding domains connected to domains that activate T cells could overcome tolerance by allowing T cells to respond to cell surface antigens. The review by Biagi and coworkers summarises the most recent advances in leukaemia immunotherapy as far as gene manipulation of donor immune cells [7]. The insertion of artificial CARs inside the gene results in the generation of T lymphocytes that produce significant tumor cell lysis. This approach seems promising especially in improving the outcome of relapsed or refractory acute lymphocytic leukaemia in children. Unfortunately, several adverse effects may occur. Data from ongoing clinical trials will shed further light on this important issue.

The quality of life of children with cancer can be affected by the experience of cancer-related pain, treatment-related pain, procedural pain, and long-term chronic pain, and the consequences may be permanent. Treatment-related pain and procedural pain are often reportedly the most painful experiences related to their illness. Procedural pain treatment is therefore now considered essential. This study conducted by Po and coworkers from the University of Padua and G.B. Rossi Hospital, Verona, Italy, reports data from a wide national survey obtained through a questionnaire that investigates the beliefs of operators on the painfulness of invasive procedures and levels of pain management in a group of Italian Pediatric Hematology-Oncology Centers [8]. Their findings confirm the overall increasing attention to pain in children: invasive diagnostic-therapeutic procedures are considered painful by all healthcare professionals involved, although pain management is generally considered good.

## Infectious diseases

Breastfeeding has a major impact on cytomegalovirus (CMV) epidemiology. Viral DNA and RNA can be easily isolated from cells and fat-free breast milk. Several recent studies described low transmission rates and mostly asymptomatic infected neonates using frozen milk. The study conducted by Chiavarini et al evaluates the rate and clinical expression of CMV infection transmitted through breast milk in preterm infants and analyses the safety of the freezing treated breast milk [9]. The authors included fifty-seven preterm infants and their CMV seropositive mothers. Fresh breast milk samples were collected from 1st to 9th postpartum weeks. Both fresh breast milk and 72, 96, 120 hours frozen samples were examined for the presence of CMV. Urine samples were also tested. Seventy percent of the mothers showed reactivation of the infection, and breast milk during the six weeks postpartum was CMV-positive. However, only one infant was infected by CMV. Authors conclude that freezing breast milk at −20°C and pasteurization may respectively reduce or eliminate the viral load. Future research is necessary to create a new procedure for gentle viral inactivation of seropositive breast milk in order to prevent CMV transmission in preterm infants.

Escherichia coli is responsible for 60 to 80% of community acquired urinary tract infections in infants and children. Drug management is usually initiated on an empiric basis, but increasing concern on antimicrobial resistance has been raised in the past years. Coracciolo et al [10] conducted a 3 year retrospective study which analyzed the susceptibility patterns of bacteria isolated from urinary tract coltures in 275 children aged from 2 to 36 months. Escherichia coli was isolated in 64% of the subjects and high rates of ampicillin resistance were evidenced while resistance to cotrimoxazole and coamoxyclav was less pronounced. No resistance or less than 1% of resistance was found for ceftazidime, ceftriaxone, nitrofurantoin and gentamycin. These results support the recommendations of the Italian Society of Pediatric Nephrology to initially manage children affected by community acquired urine tract infections with either oral coamoxyclav or parental ceftriaxone.

The bacterial spectrum for early-onset neonatal sepsis (EONS) varies in different countries and a continued surveillance is mandatory due to the temporal changes in causative organisms and in their antibiotic susceptibility. An early initiation of appropriate antibiotic therapy is fundamental in improving outcome and morbidity in these patients. Bhat and coworkers [11] conducted an audit of positive blood coltures obtained from neonates with EONS over a 7 year period indicating that Gram-negative species continue to be the predominant causative organisms. Pseudomonas and Klebsiella played a major role while Actinobacter, Staphylococci and E. Coli contributed to the rest. A cause of concern was the low susceptibility to commonly used antibiotics such as ampicillin. The initial empirical choice of cefotaxime in combination with amikacin appeared to be the rational choice for the given cohort.

Monitoring body temperature is an important clinical procedure since many conditions present with fever. Rectal temperature represents the gold standard in most clinical settings, but the oral cavity and axilla have also been traditionally used for temperature measurement using a mercury-in-glass thermometer. Studies have shown that oral temperature values are closely approximated to those taken rectally. However, oral temperature measurement cannot be used effectively in children under 5 years of age due to lack of cooperation and difficulty in ensuring appropriate mouth seal. The study conducted by Edelu et al [12] aimed at analyzing sensitivity, specificity and predictive temperature values using an infrared tympanic thermometer (IRTT) in oral mode compared to rectal temperatures measured in 400 febrile and 400 afebrile children. The mean temperature taken with the IRTT in oral mode was significantly lower than the mean temperature taken with the rectal mercury-in-glass thermometer. Therefore, IRTT used in oral mode may not be reliable in estimating “core” body temperature in children under 5 years of age, but with a fairly good sensitivity (87.3%) and specificity (96.5%) along with other advantages such as short duration of measurement, convenience and safety, may be useful in fever screening in a busy setup.

## Otolaryngology

Epidemiological studies continue to document a high prevalence of hearing impairment in newborns. Ghirri P et al [13] reviewed the database of newborn hearing screening in Pisa (Italy). Eighty-four out of 8113 neonates (1,04%) failed both transient evoked oto-acoustic emissions (TEOAE) and automatic auditory brainstem response (AABR) tests. Seventyeight of them underwent further investigations. Fortyfour ( 0,54%) patients showed false positive results while 34 (4,2%) neonates had a diagnosis of hearing impairment. Eight (0.99%) patients had unilateral hearing loss, while 26 patients (3,2%) had bilateral hearing impairment.

Childhood hearing impairment is a world-wide problem. Rajendran V et al [14] pointed out that delayed postural development and motor development are common features in profoundly deaf children. The focus of evaluation and treatment for these children is primarily based on language development. Prompt interventions are crucial for improving balance and motor performance.

## Pharmacotherapy

The European Network of Paediatric Research at the European Medicines Agency is a network of research networks, investigators and centres with recognised expertise in performing clinical studies in children. It aims to foster high-quality ethical research on quality, safety and efficacy of medicines to be used in children. It does this through networking and stakeholder collaboration with members from within and outside the European Union. The Family Pediatricians Medicines for Children Research Network was established in Italy in 2003 with the aim of developing competence, infrastructure, networking and education for pediatric clinical trials. The network, consisting of twenty Pediatric Regional Networks has progressed very well and has achieved valuable improvements concerning the conduct of pediatric clinical trials. Furthermore, ad hoc training programs have incremented knowledge about clinical trials among Family Pediatrician Investigators in Italy. The commentary by Ettore Napoleone [15] highlights that networking is necessary to build competence, to make co-operation easier and to avoid duplication of studies in children.

## Respiratory tract illnesses

Group A beta-hemolytic streptococcus is the most common bacterial cause of acute pharyngitis. Although children infected will recover clinically without antibiotics, treatment is recommended in order to prevent acute rheumatic fever and suppurative complications, accelerate resolution of clinical signs and symptoms, and prevent transmission to close contacts. The review by Regoli et al from the Department of Sciences for Woman and Child’s Health at the University of Florence, Italy, discusses the main aspects related to diagnosis and indications for treatment in Streptococcal pharyngitis [16]. Since the number of patients needed to treat (NTT) to prevent one complication after upper respiratory tract infections is over 4000, clinicians should reserve antimicrobial treatment only to well-selected cases. Oral amoxicillin is confirmed as the most effective drug. It is also very well accepted because of palatability. Macrolides should be reserved to the cases of proved allergy to β-lactams. Cephalosporins can be used in patients allergic to penicillin and have also been proposed to treat relapses.

Ambulatory health care services are important for maintaining good health in children by providing preventive services and treatment of illness and injuries. The epidemiological research conducted by Liao and coworkers from the Department of Pediatrics, Taichung, Taiwan [17] evaluates ambulatory visits of children in Taiwan by analyzing a large set of representative data from the national database, categorized according to principal diagnosis, age, and different physician specialties from 2000 to 2009. Airway diseases were the most common system diseases (approximately 50%) in all age groups. Dental and eye problems were also among the most frequent requests for consultations. The study may contribute to create a baseline data for adequately addressing the special health care needs, and their future changes, of children in Taiwan. Continued monitoring of the different use of ambulatory care among children are needed, particularly in view of the possible impact on the health care system based on these differences.

Both asthma and obesity have become more common in affluent societies during the past decades, and a correlation between the conditions has been hypothesized. The research conducted by Kajbaf et al from the Pediatric Department, Ahvaz, Iran [18] confirms that there is a strong link between pediatric obesity and asthma. The authors analyze the results of a questionnaire obtained from 903 children aged 7 to 11 years to identify patients currently suffering from asthma. Body mass index was used as a measure of adiposity. The current prevalence of wheezing among obese and overweight children was 68.7% and 37%, respectively. A statistical association between obesity and current prevalence of wheezing, night cough, and exercise-induced wheezing was evidenced. Obesity and overweight were not associated with eczema and allergic rhinoconjunctivitis, leading to the conclusion that the pathophysiology of asthma in obese and overweight children is not mediated by allergy. The parallel trends in the increase in asthma and obesity may indicate a potential link between these two conditions.

Association with other diseases or syndromes has previously been reported in cystic fibrosis, the most paradigmatic example of chronic suppurative lung disease in children. The better clinical status and improved survival rate of patients with is due to the development of therapeutic strategies that are based on insights into the natural course of the disease. Patients benefit from treatment at a specialized cystic fibrosis center with a dedicated multidisciplinary team that emphasize on frequent visits, periodic testing, and monitoring adherence to therapy. Co-morbidity may be a challenge for clinicians, who have to face more severe problems. Spinelli and coworkers from the cystic fibrosis Centre of Brescia, Italy, describe a child with cystic fibrosis and 18q distal deletion syndrome [19]. This is a novel finding. The case report underlines that the work of a multidisciplinary therapeutic team is mandatory to improve the treatment of severe and complicated cases of cystic fibrosis.

Up to 20% of patients requiring mechanical ventilation suffer from difficult weaning (the need of more than 7 days of weaning after the first spontaneous breathing trial). Muscular training is an important component of weaning protocols. The cases reported by Caprotta et al from the Pediatric Intensive Care Unit, Hospital de Trauma y Emergencia Dr. Federico Abete, Malvinas Argentinas Buenos Aires, Argentina, underlines how difficult weaning can be after prolonged mechanical ventilation in patients with severe respiratory viral infection [20]. The authors describe three children with severe respiratory insufficiency secondary to H1N1 virus infection in whom extubation failed. Disconnection from positive pressure was achieved by combining tracheostomy with an orderly, monitored and progressive muscular training plan. This type of approach may be useful in patients with difficult weaning from mechanical ventilation, but more studies are necessary to develop evidence based protocols.

## Competing interests

The authors declare that they have no competing interests.

## Author’s contributions

CC conceived the study, participated in its design carried out the literature research and helped to draft the manuscript. FS participated in the design of the study, carried out the literature research and helped to draft the manuscript. SC carried out the literature research and helped to draft the manuscript. AG carried out the literature research and helped to draft the manuscript. SB conceived the study, and participated in its design and coordination and helped to draft the manuscript. All authors read and approved the final manuscript.
